# Supplementary material for: Multi‐dataset identification of innovative feature genes and molecular mechanisms in keratoconus
Source: J Cell Mol Med. 2024 Sep 19;28(18):e70079. doi: 10.1111/jcmm.70079 (PMC11412914; doi:10.1111/jcmm.70079)
Supplement: Supplementary file 2 — Material S2. [file JCMM-28-e70079-s002.zip › 7.GSEA analysis/2.ATOH7/KEGG_VIBRIO_CHOLERAE_INFECTION.html]

Details for gene set KEGG\_VIBRIO\_CHOLERAE\_INFECTION[GSEA]

|  || Dataset | ATOH7.group.cls#h\_versus\_l |
| Phenotype | group.cls#h\_versus\_l |
| Upregulated in class | h |
| GeneSet | KEGG\_VIBRIO\_CHOLERAE\_INFECTION |
| Enrichment Score (ES) | 0.3608212 |
| Normalized Enrichment Score (NES) | 1.5187721 |
| Nominal p-value | 0.027504912 |
| FDR q-value | 0.2615466 |
| FWER p-Value | 0.747 |
Table: GSEA Results Summary

  

Fig 1: Enrichment plot: KEGG\_VIBRIO\_CHOLERAE\_INFECTION      
 Profile of the Running ES Score & Positions of GeneSet Members on the Rank Ordered List

  

| SYMBOL | TITLE | RANK IN GENE LIST | RANK METRIC SCORE | RUNNING ES | CORE ENRICHMENT || 1 | KDELR3 | na | 199 | 0.677 | 0.1199 | Yes |
| 2 | PRKACB | na | 829 | 0.474 | 0.1896 | Yes |
| 3 | ATP6V1G2 | na | 1480 | 0.388 | 0.2428 | Yes |
| 4 | PRKACG | na | 1784 | 0.361 | 0.3011 | Yes |
| 5 | PRKCB | na | 2281 | 0.324 | 0.3470 | Yes |
| 6 | CFTR | na | 4476 | 0.219 | 0.3243 | Yes |
| 7 | ADCY3 | na | 5082 | 0.198 | 0.3436 | Yes |
| 8 | ATP6V1E2 | na | 5651 | 0.181 | 0.3608 | Yes |
| 9 | ATP6V1B1 | na | 6672 | 0.152 | 0.3596 | No |
| 10 | PRKCA | na | 7938 | 0.120 | 0.3454 | No |
| 11 | PDIA4 | na | 9436 | 0.091 | 0.3192 | No |
| 12 | ATP6V0E2 | na | 10518 | 0.073 | 0.3016 | No |
| 13 | SEC61A1 | na | 10595 | 0.072 | 0.3128 | No |
| 14 | ATP6V1C2 | na | 10800 | 0.069 | 0.3197 | No |
| 15 | ARF1 | na | 11332 | 0.061 | 0.3158 | No |
| 16 | ATP6V1H | na | 11715 | 0.056 | 0.3152 | No |
| 17 | SEC61G | na | 12046 | 0.052 | 0.3153 | No |
| 18 | ATP6V1E1 | na | 12279 | 0.049 | 0.3177 | No |
| 19 | PRKACA | na | 12347 | 0.048 | 0.3247 | No |
| 20 | PRKX | na | 12697 | 0.044 | 0.3227 | No |
| 21 | ATP6V1A | na | 13508 | 0.035 | 0.3059 | No |
| 22 | ATP6V1G1 | na | 13709 | 0.033 | 0.3063 | No |
| 23 | ATP6V0C | na | 14348 | 0.027 | 0.2930 | No |
| 24 | GNAS | na | 14632 | 0.024 | 0.2893 | No |
| 25 | ATP6V1F | na | 14695 | 0.024 | 0.2919 | No |
| 26 | ATP6V1G3 | na | 15937 | 0.013 | 0.2586 | No |
| 27 | ATP6V0A1 | na | 16474 | 0.009 | 0.2448 | No |
| 28 | SEC61B | na | 16535 | 0.008 | 0.2446 | No |
| 29 | ATP6V0E1 | na | 16834 | 0.006 | 0.2372 | No |
| 30 | ATP6V1C1 | na | 16908 | 0.005 | 0.2360 | No |
| 31 | ACTG1 | na | 16994 | 0.005 | 0.2344 | No |
| 32 | ATP6V1B2 | na | 17084 | 0.004 | 0.2326 | No |
| 33 | ATP6AP1 | na | 18125 | -0.005 | 0.2035 | No |
| 34 | ATP6V0B | na | 18789 | -0.011 | 0.1864 | No |
| 35 | ATP6V1D | na | 18877 | -0.012 | 0.1861 | No |
| 36 | KDELR2 | na | 19056 | -0.013 | 0.1835 | No |
| 37 | KDELR1 | na | 19598 | -0.018 | 0.1712 | No |
| 38 | ATP6V0D1 | na | 20156 | -0.023 | 0.1595 | No |
| 39 | ADCY9 | na | 20701 | -0.029 | 0.1491 | No |
| 40 | TJP2 | na | 22117 | -0.044 | 0.1165 | No |
| 41 | ACTB | na | 22412 | -0.047 | 0.1168 | No |
| 42 | PLCG2 | na | 24577 | -0.078 | 0.0689 | No |
| 43 | KCNQ1 | na | 24718 | -0.081 | 0.0799 | No |
| 44 | TJP1 | na | 24758 | -0.081 | 0.0939 | No |
| 45 | PLCG1 | na | 25238 | -0.090 | 0.0967 | No |
| 46 | ATP6V0A4 | na | 26338 | -0.111 | 0.0857 | No |
| 47 | MUC2 | na | 26514 | -0.115 | 0.1019 | No |
| 48 | ATP6V0D2 | na | 27974 | -0.150 | 0.0878 | No |
| 49 | PRKCG | na | 28978 | -0.178 | 0.0919 | No |
| 50 | SLC12A2 | na | 29017 | -0.180 | 0.1242 | No |
| 51 | SEC61A2 | na | 30208 | -0.218 | 0.1303 | No |
Table: GSEA details [plain text format]

  

Fig 2: KEGG\_VIBRIO\_CHOLERAE\_INFECTION      
 Blue-Pink O' Gram in the Space of the Analyzed GeneSet

  

Fig 3: KEGG\_VIBRIO\_CHOLERAE\_INFECTION: Random ES distribution      
 Gene set null distribution of ES for **KEGG\_VIBRIO\_CHOLERAE\_INFECTION**
